# Induction of senescence-associated secretory phenotype underlies the therapeutic efficacy of PRC2 inhibition in cancer

**DOI:** 10.1038/s41419-022-04601-6

**Published:** 2022-02-15

**Authors:** Liping Chu, Yuxiu Qu, Yang An, Linjun Hou, Juewan Li, Weijia Li, Gaofeng Fan, Bao-Liang Song, En Li, Liye Zhang, Wei Qi

**Affiliations:** 1 Gene Editing Center, School of Life Science and Technology, ShanghaiTech University, Shanghai, 201210 China; 2 Hubei Key Laboratory of Cell Homeostasis, College of Life Sciences, Wuhan University, Wuhan, China; 3 China Novartis Institutes for BioMedical Research, 4218 Jinke Road, Shanghai, 201203 China

**Keywords:** Targeted therapies, Senescence

## Abstract

The methyltransferase Polycomb Repressive Complex 2 (PRC2), composed of EZH2, SUZ12, and EED subunits, is associated with transcriptional repression via tri-methylation of histone H3 on lysine 27 residue (H3K27me3). PRC2 is a valid drug target, as the EZH2 gain-of-function mutations identified in patient samples drive tumorigenesis. PRC2 inhibitors have been discovered and demonstrated anti-cancer efficacy in clinic. However, their pharmacological mechanisms are poorly understood. MAK683 is a potent EED inhibitor in clinical development. Focusing on MAK683-sensitive tumors with *SMARCB1* or *ARID1A* loss, we identified a group of PRC2 target genes with high H3K27me3 signal through epigenomic and transcriptomic analysis. Multiple senescence-associated secretory phenotype (SASP) genes, such as *GATA4*, *MMP2/10*, *ITGA2* and *GBP1*, are in this group besides previously identified *CDKN2A/p16*. Upon PRC2 inhibition, the de-repression of SASP genes is detected in multiple sensitive models and contributes to decreased Ki67+, extracellular matrix (ECM) reorganization, senescence associated inflammation and tumor regression even in *CDKN2A/p16* knockout tumor. And the combination of PRC2 inhibitor and CDK4/6 inhibitor leads to better effect. The genes potential regulated by PRC2 in neuroblastoma samples exhibited significant enrichment of ECM and senescence associated inflammation, supporting the clinical relevance of our results. Altogether, our results unravel the pharmacological mechanism of PRC2 inhibitors and propose a combination strategy for MAK683 and other PRC2 drugs.

## Graphical abstract

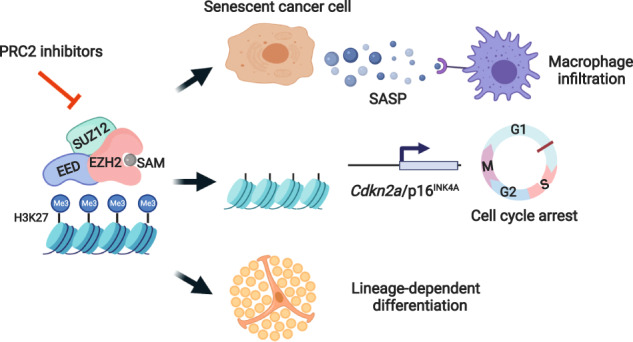

## Introduction

Polycomb Repressive Complex 2 (PRC2) is the sole histone H3 lysine 27 (H3K27) methyltransferase identified from yeast to mammals. Through binding to promoter and catalyzing the formation of trimethylated H3K27 (H3K27me3), PRC2 represses transcription of target genes and plays critical roles in embryonic development and adult stem cell maintenance in multiple lineages [1, 2]. The core PRC2 complex is composed of three subunits: catalytic subunit EZH2 or EZH1, scaffolding subunit SUZ12 and EED that allosterically activates EZH2 upon binding to H3K27me3 [3, 4]. The aberrant expression or mutation of PRC2 components has been identified in multiple diseases, especially cancers [5, 6]. Gain-of-function (GoF) mutations of EZH2 have been reported in B-cell lymphoma, melanoma and other cancers [1]. Knock-in of the GoF mutation EZH2-Y641F in mouse led to the occurrence of spontaneous lymphoma, demonstrating its onco-driver activity [7]. In addition, PRC2 epigenetically counteracts the SWI/SNF complex, which mobilizes nucleosomes to facilitate transcription. Somatic loss of SWI/SNF subunits in cancer, such as *SMARCB1* and *SMARCA4*, lead to the functional dependence on PRC2 [8]. Knockout of *SMARCB1* in mice causes tumor formation with 100% penetrance, and these tumors were highly dependent on EZH2 [9, 10].

EZH2 and EED inhibitors have been discovered and demonstrated efficacy in multiple cancer models [11–14]. EZH2 inhibitors target EZH2 directly, while EED inhibitors bind EED and inhibit PRC2 allosterically. We previously reported the discovery of EED inhibitor EED226 [12], and the optimized inhibitor MAK683 is in clinical development (NCT02900651) [15]. The EZH2 inhibitor EPZ6438 has been approved for treatment of follicular lymphoma and epithelioid sarcoma [16, 17]. Mechanistically, EZH2 depletion/inhibition de-represses cell cycle inhibitor p21^CIP1/WAF1^ (p21 for short) encoding gene *CDKN1A* and induces apoptosis in lymphoma [18–20]. Similarly, p16^INK4A^ (p16 for short) encoding gene *CDKN2A* is a target of EZH2/PRC2 [21–23] in fibroblast, mesenchymal stem cell (MSC), pancreatic beta-cell and certain cancers [24–30]. However, it is unclear whether p16 de-repression is essential or sufficient for the antitumor efficacy of PRC2 inhibitors.

Cellular senescence is a heterogenous stress response, in which cell cycle is permanently arrested but cell is metabolically alive [31]. Different stresses, such as telomere shortening, oncogene expression, epigenetic perturbation, or anti-cancer therapies, may trigger senescence. Although there aren’t senescence-unique markers, senescent cells exhibit multiple characteristics [32]. These include irreversible cell cycle arrest by expression of p16 and/or p21, appearance of senescence-associated beta galactosidase (SA-β-gal) activity, and the release of chemokines, growth regulators and extracellular matrix (ECM) components and proteases, termed senescence-associated secretory phenotype (SASP) [31, 33]. Among them, SASP is critical for senescence local spreading, but not well-characterized as SASP factors often vary depending on cell lineage and senescence inducers [34]. Simultaneous detection of multiple features confirms the occurrence of cellular senescence.

Here, we focused on the molecular pharmacological mechanism of PRC2 inhibitors and dissected the critical target genes induced by EED inhibitor MAK683 in sensitive cancer cells deficient in *SMARCB1* or *ARID1A*. PRC2 inhibition by MAK683 induces de-repression of not only *CDKN2A*/*p16*, but also *GATA4, HLA-B, MMP2/10, ITGA2* and *GBP1* with high H3K27me3 around promoters. As p16 KO does not hamper the de-repression of SASP genes, they are independently regulated by PRC2. The de-repression of *HLA-B, ITGA2* and other SASP genes by MAK683 potentiates senescence associated inflammation and macrophage infiltration and contributes to the full efficacy of PRC2 inhibitors.

## Results

### MAK683 is a highly potent PRC2 inhibitor and blocks cancer cell proliferation

**Fig. 1:**
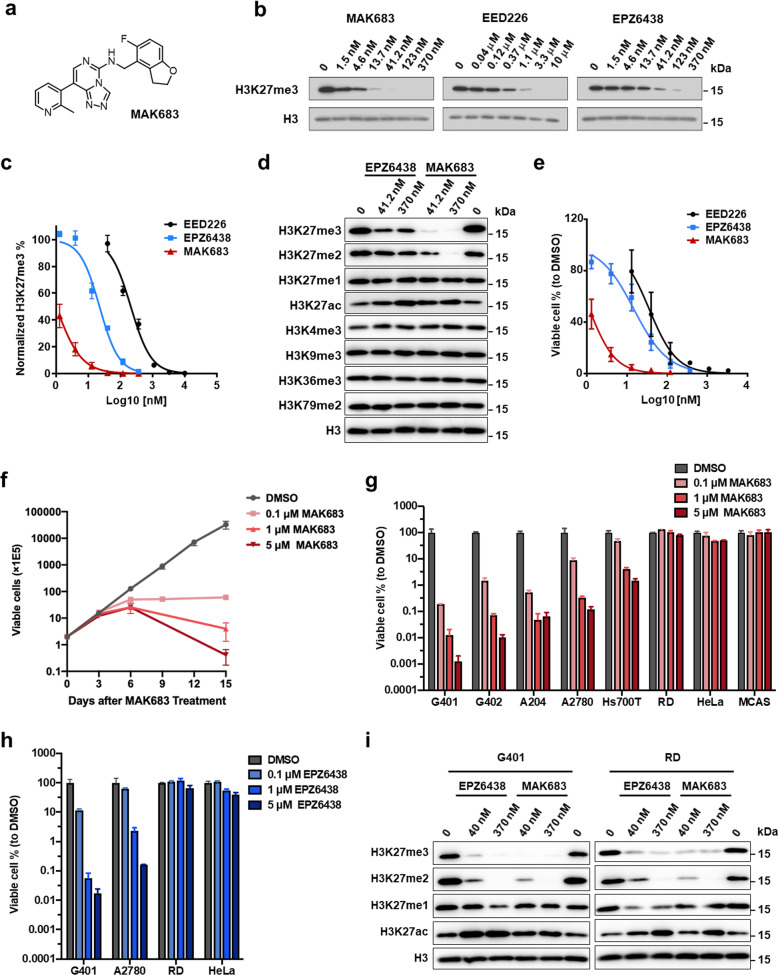
MAK683 is a potent PRC2 inhibitor blocking proliferation of multiple cancer cells. **a** Chemical structure of MAK683. **b** Inhibition of cellular H3K27me3 dose-dependently by MAK683, EED226 and EPZ6438 in HeLa cell after 72 hr of treatment. **c** Quantification of data in (**b**) and two other repetitive experiments and fit into the dose-responsive curves of H3K27me3 inhibition (*n* = 3; mean ± s.d.). IC50s were calculated using PRISM. The IC50s for MAK683, EED226 and EPZ6438 are 1.014 nM, 209.9 nM, and 22.47 nM respectively. **d** Western blots showing modulation of histone H3 methylations by EPZ6438 and MAK683 using HeLa cell lysates treated with indicated concentrations of EPZ6438 and MAK683 for 72 h. **e** Dose-dependent inhibition of the proliferation of WSU-DLCL2 (EZH2-Y641F) cell after 9 days of treatment with the indicated compounds at 6-dose points. Viable cells were counted every 3 days in the presence of the indicated compounds and results were plotted and IC50s were calculated using PRISM (*n* = 3; mean ± s.d.). The IC50s for MAK683, EED226, and EPZ6438 are 1.153 nM, 35.86 nM and 14.86 nM, respectively. **f** Proliferation of G401 cells. Viable cells were counted every 3 days in the presence of MAK683 at the indicated concentrations, and results were plotted on a logarithmic scale (*n* = 3; mean ± s.d.). **g** Inhibition of the proliferation of multiple cells including G401, G402, A204, A2780, Hs700T, RD, HeLa, and MCAS after 15 days of MAK683 treatment. Viable cells were counted every 3 days in the presence of MAK683 at the indicated concentrations, and results were normalized to the control samples (DMSO as 100%) of respective cells and then plotted on a logarithmic scale (*n* = 2 or 3; mean ± s.d.). **h** Inhibition of the proliferation of G401, A2780, RD, and HeLa after 15 days of EPZ6438 treatment. Viable cells were counted every 3 days in the presence of EZP6438 at the indicated concentrations, and results were expressed similarly as in (**g**). (*n* = 2; mean ± s.d.). **i** Western blots showing modulation of histone H3K27 methylations and acetylation by EPZ6438 and MAK683 in G401 and RD cells treated with indicated concentrations of compounds. All experiments were repeated for more than two times.

The PRC2 inhibitors MAK683 and EED226 bind EED directly and inhibit both EZH1/PRC2 and EZH2/PRC2 (Fig. 1a) [12, 15]. We acquired these two compounds together with EPZ6438, an FDA-approved EZH2 inhibitor, and compared them in cellular experiments. First, they all decreased H3K27me3 dose-dependently in HeLa cell. The IC50 of MAK683 was 1.014 nM, the lowest among them (Fig. 1b, c). The IC50s of EED226 and EPZ6438 were 209.9 nM and 22.47 nM, respectively. Their selectivity is high, as only methylation and acetylation of H3K27 were altered (Fig. 1d and Supplementary Fig. 1a). MAK683 removed more H3K27me2/me3 comparing with EPZ6438 at the same concentration, likely due to its EED-binding mechanism or higher potency. Then, they were tested in anti-proliferation of lymphoma cell WSU-DLCL2 (WSU) with GoF mutation EZH2-Y641F [11, 35]. These compounds inhibited the proliferation of WSU with IC50s of 35.86 nM for EED226, 14.86 nM for EPZ6438 and 1.153 nM for MAK683 (Fig. 1e).

We then examined the anti-proliferation of MAK683 in a panel of cancer cells. G401, G402 and A204 are malignant rhabdoid tumor (MRT) cells with *SMARCB1* deficiency [22]. They were sensitive to MAK683 treatment (Fig. 1f, g). Ovarian cancer cell A2780 with *ARID1A*-deificiency and pancreatic cancer cell Hs700T with *SMARCA4*-deficiency were also moderately sensitive to MAK683. EPZ6438 similarly inhibited the proliferation of A2780, while RD, HeLa and MCAS cells were not sensitive to PRC2 inhibition by MAK683 or EPZ6438 (Fig. 1g, h) [36]. The growth plot of these sensitive cells presented a slow inhibition by MAK683, a typical response to epigenetic inhibitors (Supplementary Fig. 1b-d) [11, 14, 22]. The proliferation of HeLa and RD was not affected at all, even though the H3K27me3 level was dramatically decreased by MAK683 or EPZ6438 (Fig. 1b, g, and i). It was hypothesized that the increase of H3K27ac after H3K27 methylation removal is an important factor affecting sensitivity to PRC2 inhibitors [4, 37]. Here, the increase of H3K27ac was observed in both sensitive G401 and refractory RD (Fig. 1i). So, adding H3K27ac at the right position may be critical.

Together, EPZ6438 and MAK683 are potent PRC2 inhibitors, and they inhibit proliferation of tumor cells with EZH2-GoF mutation or deficiency of SWI/SNF complex. We mainly used MAK683 for PRC2 inhibition in the following studies.

### A signature of high H3K27me3 marks genes derepressed by PRC2 inhibition

**Fig. 2:**
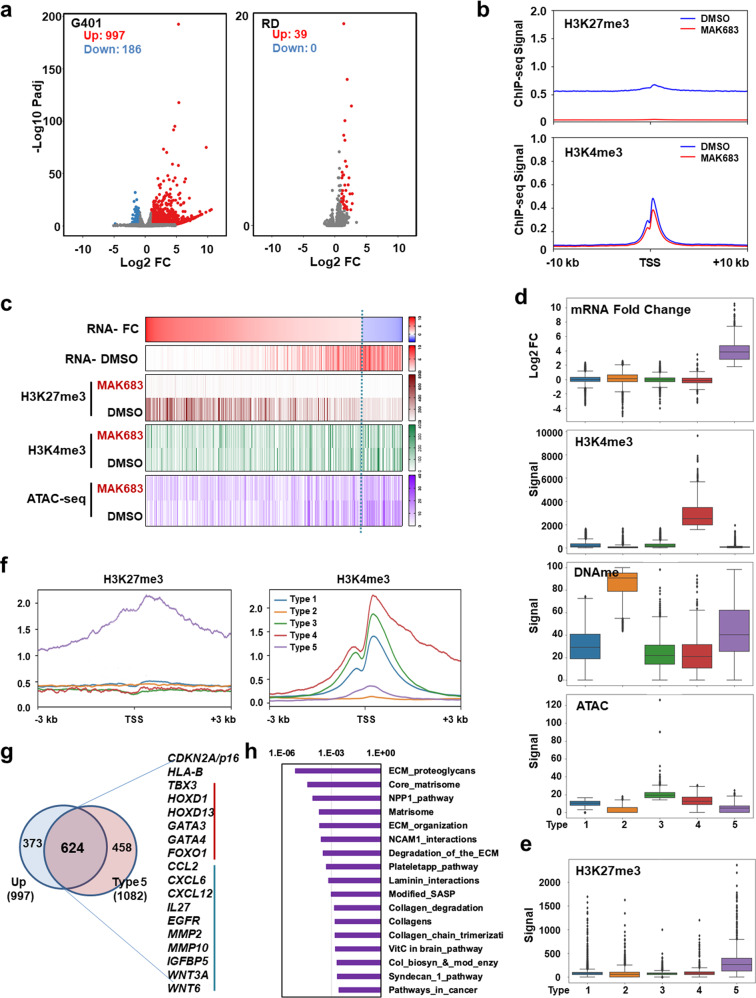
Multi-omics integrated analysis suggests that H3K27me3-high genes are direct PRC2 target genes derepressed by PRC2 inhibition. **a** Volcano plot showing the differential expression genes (DEGs) of G401 and RD cell after DMSO, MAK683 treated for 3 days. Red shows up-regulated after MAK683 treatment (log2 fold change ≥ 1 and *p* adjust ≤ 0.05) and blue indicates down-regulated after MAK683 treatment (log2 fold change ≤ −1 and *p* adjust ≤ 0.05) unless otherwise noted. **b** Composite H3K27me3 profile and H3K4me3 profile around transcripts start sites (TSS) in G401 cell. **c** Heat map of log2 mRNA fold change levels, mRNA expression levels, H3K27me3 modification levels, H3K4me3 modification levels and Chromatin accessibility levels in G401 cell. **d** Box plots of log2 mRNA fold change levels, H3K4me3 modification levels, DNA methylation levels and chromatin openness levels between different clustering types in G401 cell. The whiskers of the box plot extend to 1.5 times the interquartile range unless otherwise noted. **e** Box plots of H3K27me3 modification levels between different clustering types in G401 cell. **f** Composite H3K27me3 profile and H3K4me3 profile around TSS in different clustering types in G401 cell. **g** Venn diagram showing the overlap of type 5 genes from clustering (1082 genes) with upregulated DEGs from RNA-seq analysis (997 genes). The genes lie in both classes include multiple typical PRC2 target genes in ES cells (indicated by red line) and senescence-associated secretory factors (indicated by blue line). **h** Pathway enrichment analysis of the 624-gene class from the (**g**).

Next, we seek to understand what happens in sensitive tumor after PRC2 inhibition. We performed RNA-seq on three sensitive cells G401, G402 and A2780, and one refractory cell RD. Hundreds of genes exhibited upregulation by MAK683 treatment in the sensitive cells, while only 39 genes showed mild increase in RD (Fig. 2a and Supplementary Fig. 2a). So, transcriptional activation is a specific event in sensitive cells. To further dissect the epigenomic changes associated with PRC2 inhibition, we selected G401 for profiling studies including Whole Genome Bisulfite Sequencing (WGBS), H3K4me3 and H3K27me3 chromatin immunoprecipitation-sequencing (ChIP-seq) and assays for transposase-accessible chromatin (ATAC)-seq. Massive genome-wide depletion of H3K27me3 signal was observed (Fig. 2b), while overall DNA methylation pattern, H3K4me3 signal and chromatin opening around transcription start site (TSS) were not significantly changed (Fig. 2b and Supplementary Fig. 2b, c).

When differentially expressed genes (DEG, Log2Fold Change > = 1 and *p adj* < 0.05) were laid out by fold change (FC), there was a trend that the highly upregulated genes have higher H3K27me3 signal in DMSO sample (Fig. 2c). Next, to find out the epigenetic features of these PRC2 inhibition-upregulated genes, we performed unsupervised clustering using mRNA expression-FC, DNA methylation, H3K4me3 and ATAC-seq data. This analysis gave 5 different gene types, each represented a different epigenetic characteristic (Fig. 2d). For example, type 2 showed high DNA methylation, type 4 showed high H3K4me3 level, while type 5 enriched most of the upregulated genes (Fig. 2d). Interestingly, the type 5 genes had significantly higher H3K27me3 signal than other types at basal state (Fig. 2e). By examining the average distribution of H3K27me3 and H3K4me3 from −3 kb to +3 kb, we found H3K27me3 of type 5 genes showed a clear peak around TSS (Fig. 2f). As the clustering analysis used G401 data, we examined RNA-seq data of A2780 and found type 5 genes were also significantly upregulated in MAK683-treated A2780 (Supplementary Fig. 2d). So, the type 5 genes are likely direct targets of PRC2. Overlapping of type 5 genes with the upregulated DEGs in G401 gave a list of 624 genes, which included many well-characterized PRC2 targets such as *CDKN2A/p16*, *HLA-B*, *TBX3*, *HOXD1*, *GATA3*, and *GATA4* (Fig. 2g). Majority of these genes (578 out of 624) were also H3K4me3 positive (Supplementary Fig. 2e, f). In the enrichment analysis, the top 5 pathways highlighted ECM and proteoglycan. The enriched genes included *COL4A1*, *COL4A2*, *COL4A4*, *COL4A5*, *FBN2*, and *LAMB1*, which are typical basement membrane markers (Fig. 2h) [38]. Considering PRC2 represses differentiation in development [39] and the origin of MRT is neural crest [40], these enriched pathways and genes suggest PRC2 inhibition in tumor induces epithelial differentiation and ECM reorganization.

### PRC2 inhibition induces multiple features of cellular senescence in sensitive tumors

**Fig. 3:**
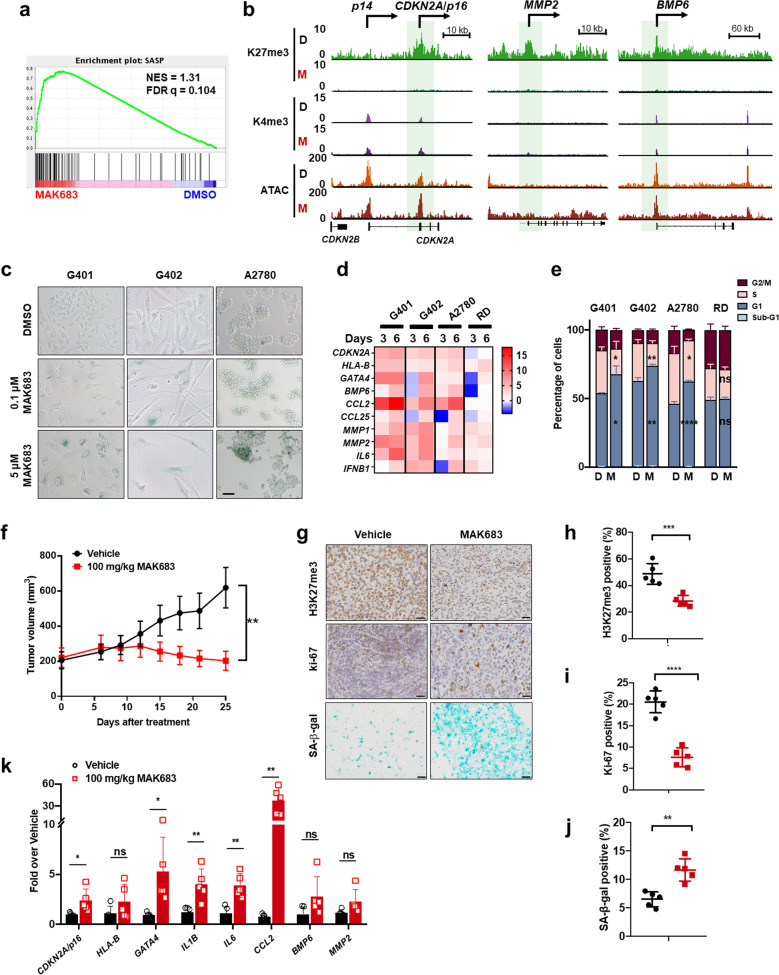
Senescence induction is a shared phenotype in multiple MAK683-responsive solid cancer cells in vitro and in vivo. **a** GSEA of RNA-seq data from G401 cell with DMSO or MAK683 treatment as shown in Fig. 2a revealing the enrichment of senescence-associated secretory phenotype (SASP). **b** H3K27me3 and H3K4me3 ChIP-seq tracks and ATAC-seq tracks at *p16/CDKN2A*, *MMP2* and *BMP6* loci in G401 cells treated with DMSO or MAK683. Green highlights indicated genomic regions around TSS. **c** Representative senescence-associated β-galactosidase staining (SA-β-gal, blue) of G401, G402, A2780 and RD cells treated with DMSO or MAK683 at the indicated concentrations for 9 days. RD cell is not sensitive to MAK683 treatment and is negative for SA-β-gal. Scale bars represent 50 μm. Representative images of more than two independent experiments in the indicated cells. **d** Heat map showing the expression fold changes of the indicated genes in MAK683 treated G401, G402, A2780 and RD cells assessed by RT-qPCR. These genes were selected based on Fig. 2f and Supplementary Fig. 3c (SASP heat map). **e** Bar graph presenting the cell cycle changes analyzed by FACS after treatment with DMSO or 3 μM of MAK683 for 3 days (*n* = 3; mean ± s.d.). The *P* values were determined by Multiple *t* test (*, *p* < 0.05; **, *p* < 0.01; ****, *p* < 0.0001; ns, not significant). **f** Growth curve of subcutaneous G401 xenograft tumors in mice treated with MAK683 in a suspension formulation through oral administration (po) once daily (qd) for 25 days. On day 25, tumor tissues were collected at 4 h post treatment for molecular analysis. Data are shown as mean ± s.e.m. (*n* = 6; **, *p* < 0.01). **g** Representative H3K27me3, Ki67 and SA-β-gal staining of the tumor xenograft collected from the study in (**f**). Scale bars, 20 μm. Quantification of the H3K27me3 positive (**h**), Ki-67 positive (**i**) and SA-β-gal positive (**j**) percentage. Mean ± s.d. of positively stained cell percentage (Ki67 and H3K27me3) or positively stained area (SA-β-gal) are shown. *P* values were determined by Multiple *t* test (**, *p* < 0.01; ***, *p* < 0.001; ****, *p* < 0.0001). **k** Quantitative PCR analysis of gene expression changes in tumor samples after vehicle or MAK683 dosing. RNA was harvested from the tumor xenograft collected from the study in (**f**) (*n* = 6; mean ± s.d.). (*, *p* < 0.05; **, *p* < 0.01). All experiments were repeated for more than two times.

As upregulated genes by PRC2 inhibitors are likely the drivers for tumor sensitivity, we focused on the transcriptome data and performed Gene Set Enrichment Analysis (GSEA). “Regulation of senescence pathway” and “SASP” were significantly enriched in MAK683-treated samples (Fig. 3a and Supplementary Fig. 3a) [41]. There were 48 genes commonly upregulated in G401, G402 and A2780 (Supplementary Fig. 3b), and *CDKN2A/p16* and *HLA-B* were at the top of this gene heatmap (Supplementary Fig. 3c). Many genes exhibiting the signature of high H3K27me3 and positive H3K4me3 decorations on promotor were clustered in type 5 gene group (Fig. 3b). Surprisingly, in addition to *CDKN2A/p16*, many of these 48 genes were annotated as upregulated genes in cellular senescence (red asterisk in Supplementary Fig. 3c, SeneQuest database) [32].

Usually, multiple features of cellular senescence co-exist upon senescence induction [32]. Indeed, in addition to SASP upregulation, MAK683 treatment enhanced SA-β-gal activity in sensitive tumor cells (Fig. 3c and Supplementary Fig. 3d, e). *GATA4* is a transcriptional factor linked to senescence and SASP [42], and it is also a PRC2 target gene in ES cells [43]. Using quantitative PCR (qPCR), we confirmed the strong upregulation of *CDKN2A/p16*, *HLA-B, GATA4* and other SASP genes, including *BMP6*, *CCL2*, *CCL25* and *MMP2* in multiple sensitive cells (Fig. 3d). In MAK683-induced senescence, we also observed significant G0/G1 cell cycle arrest and increase of p16 protein (Fig. 3e and Supplementary Fig. 3f). Moreover, the *CDKN2A/p16* promoter exhibited high H3K27me3 signal in ChIP-qPCR of G401, which strongly diminished upon MAK683 treatment. Meanwhile, H3K27ac and H3K4me3 at the same regions were increased (region 2 and 3, Supplementary Fig. 3g), confirming PRC2 directly regulates *CDKN2A/p16*.

Next, we went on to confirm the cellular observation in G401 xenograft. MAK683 showed good efficacy in tumor growth inhibition (Fig. 3f). By 25 days treatment, the tumor volume T/C% was 30.55%, and the TGI reached 67.20%. MAK683 at 100 mg/kg daily was safe, as the animals’ body weight was unaffected (Supplementary Fig. 3h). H3K27me3 was significantly decreased in MAK683-treated tumors, assuring PRC2 inhibition (Fig. 3g, h). MAK683-treated tumors exhibited significantly reduced Ki-67 and increased SA-β-gal activity (Fig. 3g–j), in agreement with the cellular observation. The mRNA level of *CDKN2A/p16* and protein levels of p16 were both significantly upregulated (Fig. 3k and Supplementary Fig. 3i). Moreover, the typical SASPs including *GATA4, CCL2, IL6* and *IL1B* were all significantly upregulated, and *HLA-B*, *BMP6* and *MMP2* showed the trend of upregulation (Fig. 3k). So, multiple features of senescence were induced by PRC2 inhibition in tumor xenograft.

### SASP gene-upregulation by PRC2 inhibition is independent of p16

**Fig. 4:**
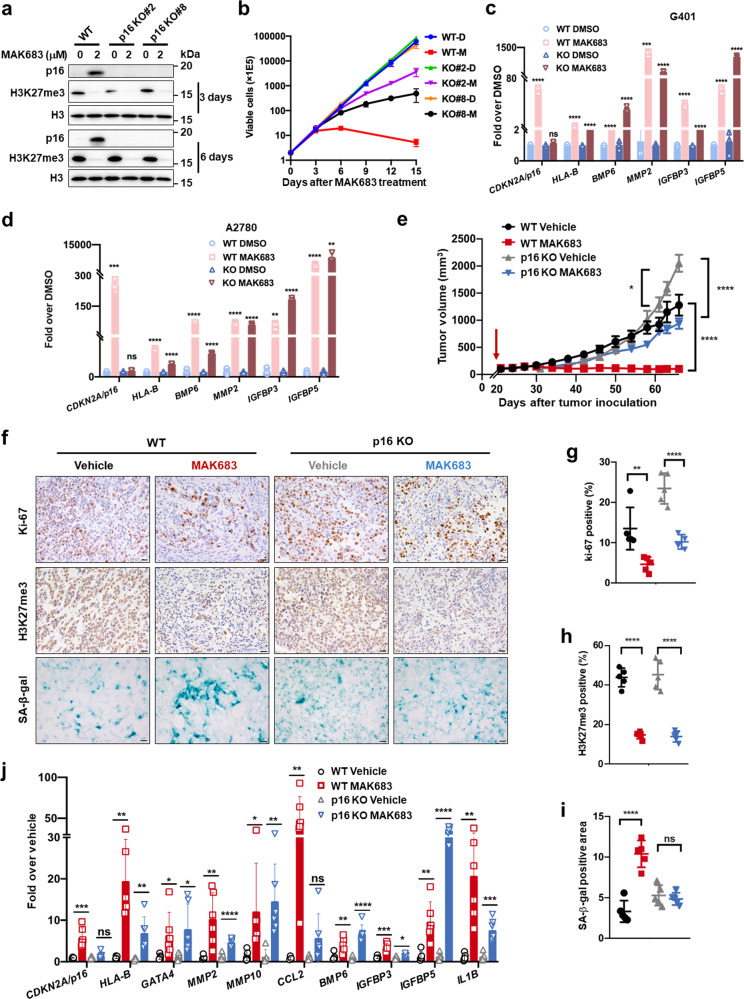
SASP upregulation by PRC2 inhibition is independent of p16. **a** Western blotting analysis showing the successful knockout of p16 in KO#2 and KO#8 cell clones, and the upregulation of p16 protein levels in MAK683 treated G401 cell are shown as positive control. Inhibition on H3K27me3 by MAK683 in all three cells is similar. **b** Proliferation of G401, p16 KO#2 and p16 KO#8 cells. Viable cells were counted every 3 days in the presence of MAK683 at 1 μM, and results from three repeat experiments were plotted on a logarithmic scale. Quantitative PCR analysis of the gene expression changes in G401 or p16 KO#2 cells (**c**) and A2780 or p16 KO#23 cells (**d**) with DMSO or MAK683 at 3 μM for 6 days (mean ± s.d.). Out of the two p16 KO clones G401, KO#2 cell is used in the studies of the following panels and labeled as p16 KO. **e** Antitumor activity of MAK683 in a suspension formulation in subcutaneous G401 and p16 KO xenograft tumors after continuous treatment for 46 days (*n* = 6; mean ± s.e.m.). The red arrow indicates the time point dosing starts. On day 46, tumor tissues were collected at 4 h post treatment for molecular analysis. **f** Representative Ki67, H3K27me3, and SA-β-gal staining of the tumor xenograft collected from the study in (**e**). Scale bars, 20 μm. Bar graphs showing the quantification of the Ki67 (**g**), H3K27me3 (**h**) and SA-β-gal (**i**) positivity. Mean ± s.d. of positively stained cell percentage (Ki67 and H3K27me3) or positively stained area (SA-β-gal) are shown. *P* values were determined by Multiple *t* test. **j** Quantitative PCR analysis of gene expression changes in G401 or p16 KO tumor samples after vehicle or MAK683 dosing. RNA was harvested from the tumor xenograft collected from the study in (**e**) (*n* = 6; mean ± s.d.). All experiments were repeated for more than two times.

*CDKN2A/p16* was considered a critical PRC2 target for proliferation blockage upon PRC2 inhibition in many cancers including nasopharyngeal carcer, breast cancer, leukemia, and ovarian cancer [44–47]. However, the requirement of p16 in these circumstances has not been studied yet. We observed the upregulation of SASPs and SA-β-gal activation in addition to *CDKN2A/p16* upregulation. To clarify their individual roles, we constructed *CDKN2A/p16* knockout (KO) G401 cells using Cas9-CRISPR method (Supplementary Fig. 4a, b). Two independent clones with confirmed genomic sequence-change and p16 protein-loss were subjected to proliferation, cell cycle and SA-β-gal analysis. The p16 deficiency did not alter the level of H3K27me3, while the p16 KO cells were partially resistant to MAK683-induced cell cycle arrest and proliferation blockage (Fig. 4a, b and Supplementary Fig. 4c). Interestingly, they also lost SA-β-gal activation by MAK683 (Supplementary Fig. 4d). However, the MAK683-induced SASP factors, such as *MMP2*, *BMP6*, *IGFBP3* and *IGFBP5*, were not affected by p16 KO (Fig. 4c). *CDKN2A/p16* KO clone in A2780 showed similar results and SASPs were still significantly upregulated by MAK683 (Fig. 4d and Supplementary Fig. 4e–h). So, the causal link between PRC2 inhibition and SASP upregulation in the sensitive cells is independent of p16.

Next, we performed in vivo xenograft study. The p16 KO G401 xenograft grew more robustly than WT xenograft (Fig. 4e, comparing the two vehicle groups). As MAK683 treatment lasted longer in this study, the treatment effectively blocked WT tumor growth (T/C% = 7.21%, TGI reached 91.25% by treatment endpoint). Furthermore, the p16 KO xenograft also responded to MAK683 treatment (T/C% = 35.81%, TGI reached 54.18% by treatment endpoint) (Fig. 4e). The Ki67 positivity correlated well with tumor size, higher in p16 KO vehicle than in WT vehicle group and was reduced by MAK683 in both WT and p16 KO tumors (Fig. 4f–h). SA-β-gal signal was increased by MAK683 treatment in WT tumors but was unchanged in p16 KO xenografts (Fig. 4f, i), which is consistent with the cellular observation (Supplementary Fig. 4d). The upregulation of classical SASPs including *MMP2*, *MMP10*, *BMP6*, *IGFBP3*, *IGFBP5* and *IL1B* was statistically significant and unaffected by p16 KO, and the same trend for *CCL2* was observed (Fig. 4j). Together, these results suggest a class of SASP genes are directly regulated by PRC2. They are upregulated by PRC2 inhibition independent of p16 and contribute to tumor repression by PRC2 inhibitors.

### PRC2 inhibition promotes tumor differentiation, senescence and immune infiltration in vivo

**Fig. 5:**
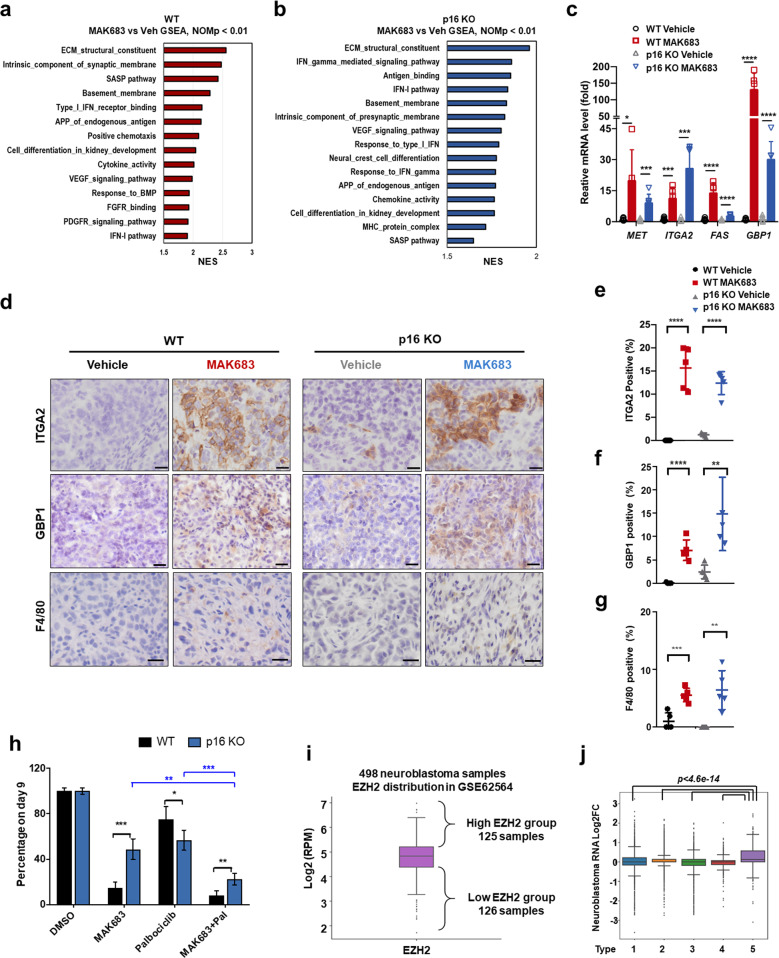
PRC2 inhibition promotes tumor cell differentiation, senescence-associated inflammation and immune infiltration in vivo. GSEA of RNA-seq data from the G401 (**a**) or p16 KO (**b**) tumor xenograft collected in the study in Fig. 4E, showing the enrichment of ECM, type I IFN and antigen processing and presentation (APP) gene signatures, and cell differentiation after PRC2 inhibition. **c** Quantitative PCR analysis of the expression changes of IFN-I response genes in G401 or p16 KO tumor samples after vehicle or MAK683 dosing. RNA was harvested from the tumor xenograft collected from the study in Fig. 4e (*n* = 6; mean ± s.d.). **d** Representative ITGA2 and F4/80 staining of G401 or p16 KO tumor xenograft after vehicle or MAK683 dosing collected from the study in Fig. 4E. Scale bars represent 20 μm. Bar graphs showing the quantification of the ITGA2 positive percentage (**e**), GBP1 positive percentage (**f**) and F4/80 positive percentage (**g**). Mean ± s.d. of positively stained cell percentage or area are shown. *P* values were determined by Multiple *t* test. **h** Inhibition of the proliferation of G401 WT or p16 KO cells by combination of MAK683 and CDK4/6 inhibitor Palbociclib treatment. Viable cells were counted every 3 days in the presence of 50 nM of MAK683, 1 nM of Palbociclib, or combo of them, and results were normalized to DMSO samples (*n* = 4; mean ± s.d.). *P* values were determined by Multiple *t* test (*, *p* < 0.05; **, *p* < 0.01; ***, *p* < 0.001). **i** Box plot of mRNA expression levels (log2 (RPM)) of *EZH2* in 498 neuroblastoma samples (GSE62564). The quarter of samples with the high level of EZH2 expression (125 samples) were designated as high EZH2 group, and the quarter of samples with the low level of EZH2 expression (126 samples) were designated as low EZH2 group. **j** Box plots of log2 mRNA fold change (FC, calculated as shown in Supplementary Fig. 5e) between different clustering types (same gene lists of every type as in Fig. 2d) in neuroblastoma data. *P* values were determined by Kruskal nonparametric test. All experiments were repeated for more than two times.

As the p16 KO xenograft still responded to MAK683, we performed RNA-seq using the samples from Fig. 4e to dissect the changes by PRC2 inhibition. Multiple pathways were significantly enriched from GSEA in MAK683-treated tumors, such as ECM constituents, synaptic membrane components and basement membrane pathways (Fig. 5a, b and Supplementary Fig. 5a, b), consistent with the results of cellular multi-omics analysis (Fig. 2h). The enrichment of pathways “neural crest cell differentiation” and “cell differentiation in kidney” likely reflected the neural crest origin of MRT [40]. Differentiation after EZH2 inhibition has been reported in lymphoma [1, 11]. Thus, differentiation induction may be a general theme after PRC2 inhibition.

Nonclassical SASP factors including ECM components have been recognized as critical players in senescence [31, 33]. SASP pathway and ECM were both significantly upregulated in MAK683-treated tumors (Fig. 5a, b). More importantly, pathways related to innate immunity were highly enriched in MAK683-treated WT and p16 KO tumors, such as type I IFN pathway, antigen processing and presentation (APP) of endogenous antigen (Fig. 5a, b and Supplementary Fig. 5a, c), confirming these functional outputs of PRC2 inhibition is independent of p16.

Many ECM, APP, IFN-I and SASP genes were type 5 genes with high H3K27me3 signal and upregulated by MAK683 treatment, including *ITGA2*, *COL4A5*, *DSG2*, *FBN1*, *FAS, GBP1* and *FRAS1* (Supplementary Fig. 5a, c and d). qPCR analysis confirmed their upregulation in MAK683-treated WT and p16 KO tumors (Fig. 5c and Supplementary Fig. 5b). Senescent MSCs upregulated ECMs included ITGA2, FBN1, COL1 and COL4 [48]. ITGA2 and GBP1 proteins were significantly increased in MAK683-treated WT and p16 KO tumors (Fig. 5d–f). Interestingly, ITGA2 was a senescence associated inflammatory factor in colorectal cancer [49, 50], while GBP1 was an interferon-responsive gene in innate immunity [51]. SASPs facilitated macrophage and other immune cell infiltration to clear senescence cells in cancer therapy and tissue repairing [52, 53]. We detected the mouse macrophages infiltration in tumor slices and found F4/80 signal was indeed high in MAK683-treated WT and p16 KO tumors (Fig. 5d, g). As p16 blocks cell cycle through inhibiting CDK4/6, combination of MAK683 and CDK4/6 inhibitor Palbociclib showed better proliferation inhibition in p16 KO cell (Fig. 5h). Altogether, PRC2 inhibition in sensitive tumor induced significant upregulation of p16, ECM and SASP factors, and promoted macrophage infiltration.

Next, we seek validation in human samples with a data set of 498 neuroblastoma cases (GSE62564) [54], as the origin of neuroblastoma was also neural crest cells [40] and neuroblastoma is sensitive to PRC2 inhibitor [55]. First, using EZH2 expression to rank samples, we identified a group of 126 samples with low EZH2 and a group of 125 samples with high EZH2 (Fig. 5i). Then we calculated gene expression FC for all genes using formula in Supplementary Fig. 5e and got 3006 up-DEGs through low EZH2 group vs high EZH2 group comparison. Further pathway analysis showed enrichment of neuronal function (green), cell-cell adhesion (brown) and interferon response pathways (red, Supplementary Fig. 5f) in up-DEGs class, which were consistent with MAK683-treated cells and xenografts (Figs. 2h, 5a, b). Meanwhile, we examined the log2FC of the 5 types of genes from clustering (Fig. 2d). Similarly, the type 5 genes showed significant higher level in neuroblastoma with low EZH2 (Fig. 5j). This is a relevant piece of evidence supporting PRC2 may regulate SASPs and IFN pathways in human neuroblastoma.

## Discussion

Deciphering the molecular pharmacological mechanisms of a target therapy is crucial for its appropriate clinical application and maximizing the benefit it may bring to patients. In this study, we addressed a fundamental question for PRC2 inhibitors, how do they work in responsive tumors. Using MAK683 as an example, our results indicate there are at least three independent molecular responses upon PRC2 inhibition and H3K27me3 loss. Firstly, *CDKN2A/p16* is transcriptionally derepressed in treated tumors. This is likely responsible for acute cell cycle arrest. Secondly, there is a differentiation program induced by PRC2 inhibition. As the origin-of-tumor for MRT is neural crest, a prominent induction of genes related to axon guidance and neuronal functions are derepressed. This effect may also be related to the bivalent genes in embryonic development. Finally, PRC2 inhibition enables the de-repression of SASP factors and ECM genes, including *GATA4*, *HLA-B*, *MMP2/10, BMP6*, *FAS, GBP1* and *ITGA2*. These gene products may enhance the antigen presentation and immune infiltration to potentiate efficacy of MAK683. In total, these responses all contribute to the antitumor efficacy of PRC2 inhibitors.

H3K27 methylation covers more than 50% of genomic region and exhibits broad distribution in cancer cells [1, 39]. However, only hundreds of genes showed transcriptional de-repression after PRC2 inhibition and H3K27me3 removal [11, 13, 56]. This contradictory observation suggest that H3K27me3 removal may only be permissive for PRC2 target activation. Indeed, studies in embryonic stem cells revealed the bivalency model, in which H3K4me3 and H3K27me3 often co-reside on the promoters of the developmental genes to transiently repress their expression [57]. When H3K27me3 is removed, H3K4me3 would quickly drive the upregulation of these genes, and therefore push forward the developmental process. Many of the type 5 genes exhibit moderate H3K4me3 signal, such as *GATA4, HLA-B*, *CDKN2A/p16*, *HOXD13, TBX3, FOXO1* and *WNT6* (Fig. 3b and Supplementary Fig. 2e, 2f). They may be the remanence of bivalent genes.

p16 is a classical senescence marker. It has been used as a label for senescent cells, and depletion of p16-positive cells in aged mice extended their healthy lifespan [58]. It is also a typical bivalent gene (Fig. 3b) [30, 57, 59], which explains why it is generally regulated by PRC2. Interestingly, we found that p16 protein correlated with SA-β-gal activity, but not SASP. The p16 KO cells did not turn on SA-β-gal with MAK683 treatment (Fig. 4f, i and Supplementary Fig. 4c). Consistently, overexpression of p16 or p21 in normal human fibroblasts induced senescent morphology and expression of SA-β-gal without SASP [60]. There are multiple possible reasons. For example, lysosomal amplification may occur in G1 of cell cycle, and senescent cells are long-arrested in G0/G1 and therefore accumulate lysosomes. Alternatively, p16 or CDK4/6 inhibition may be actively required for SA-β-gal gene *GLB1* expression with an unknown mechanism. Further investigation on the linkage between p16 and SA-β-gal would be warranted. Meanwhile, p21 was not upregulated in the RNA-seq data of MAK683 treated cells (GSE183600), and the involvement of p53/p21 axis merited further investigation.

Other than p16 and p21, there are a few PRC2 target genes reported previously in literature. For example, the MHC class I molecules are bivalent genes under the control of PRC2. PRC2 depletion or inhibition would significantly upregulate their expression in multiple cell types [61]. T helper 1-type chemokines *CXCL9* and *CXCL10* are silenced by PRC2 in ovarian and colon cancer [62, 63]. *IGFBP3* and *IGFBP5* have also been shown under the regulation of PRC2 [64, 65]. Interestingly, *HLA-B*, *CXCL9, CXCL10*, *IGFBP3* and *IGFBP5* are all senescence-upregulated genes in SeneQuest database [32]. It is conceivable that these genes may be SASP factors repressed by PRC2, consistent with our results. Tissue- and lineage-dependency is an intrinsic feature of SASP, which is also a characteristic for epigenetic marks. It is well-noted that epigenome changes dramatically during senescence and multiple epigenetic modulators are involved, such as BRD4 and MLL1 [66, 67]. Our work provides strong evidence that PRC2 belongs to this list. Furthermore, many of these genes are tumor suppressors, such as *GATA4* and *IGFBP3/5*, and may contribute to the therapeutic effect of PRC2 inhibition in tumor (Fig. 4e).

In summary, our results suggest that PRC2 inhibitors in cancer treatment induce differentiation and multiple features of senescence, including p16, ECM, and SASPs to render their anti-cancer efficacy. Senescence-induced inflammation further attracts immune infiltration for senescent cell clearance [33] (Fig. 5d, g). Along the line, combining PRC2 inhibitor with senolytic agents, such as navitoclax (BCL2 inhibitor), may provide a way to enhance tumor shrinkage and patient survival. Recently, Morel et al. reported that EZH2 depletion/inhibition upregulated IFN pathways and potentiated response to PD-1 therapy in prostate cancer [68], which may be similar for MRT and ovarian cancers. Meanwhile, the application of CDK4/6 inhibitor together with MAK683 achieved better efficacy, especially in p16 inactivated tumors (Fig. 5h). We used *SMARCB1* or *ARID1a* deficient models regardless of location. These pieces of evidence support that PRC2 inhibitors could induce therapeutic response in cancer from different body location, and the combination of PRC2 inhibitor with PD-1 therapy and/or CDK4/6 inhibitors could be a valid clinic strategy.

## Materials and methods

### Cell culture

Cells were maintained in a humidified incubator at 37 °C with 5% (vol/vol) CO_2_. G401, G402, A204 were cultured in DMEM (Invitrogen, 12100046) with 10% (vol/vol) FBS (Lonsera, S711-001S), 0.055 mM 2-mercaptoethanol (Sigma, M3148) and 1% penicillin/streptomycin. MCAS, HEK293T, A2780, Hs700T, U2OS, RD, HeLa were cultured in DMEM (Invitrogen, 12100046) with 10% (vol/vol) FBS and 1% penicillin/streptomycin. WSU-DLCL2 was cultured in RPMI1640 (Invitrogen, 31800022) with 15% (vol/vol) FBS and 1% penicillin/streptomycin. All cells are authenticated by STR profiling and tested for confirmation of mycoplasma-free. EED226, EPZ6438 and MAK683 were purchased from MCE and Selleck.

### Cell proliferation and cell cycle analysis

Cells were seeded in 6-well plates at a density of 2 × 10^5^ cells with the indicated concentrations of EPZ6438 or MAK683. Viable cell numbers were counted every 3 days up to 15 days by Vi-CELL (Beckman Coulter). For cell cycle analysis, MAK683 treated cells were fixed with pre-chilled 70% ethanol and kept at -20 degree overnight. On the second day, the cells were washed with cold PBS twice and collected by spin at 1000 rpm, digested with RNase A, then stained with 10 μg /mL Propidium Iodide in PBS solution for 30 min at room temperature. The stained cell samples were filtered through 70 μm cell strainer, and the staining signals were detected using flow cytometer (BD Fortessa). The acquired data were further analyzed by Modfit LT.

### Lentiviral infections

HEK293T cells were co-transfected with plasmid DNAs of a lentiviral vector, packaging plasmid psA2X and envelope plasmid pMD2G using Lipofectamine 2000 (Invitrogen) with the ratio of 4:3:1. The supernatant was collected 48 and 72 hr after transfection and filtered by 0.45 μM filter. The fresh filtered lentiviruses were used to infect the target cells G401 and A2780 in the presence of 8 μg /mL polybrene (Sigma). After 72 hr, GFP positive cells were sorted by flow cytometer (BD Melody). For cells infected with shRNA vectors, puromycin resistant cells were cultured under the selection of 0.7 μg/mL puromycin for 4 days before further experiments.

### CRISPR/Cas9 gene editing

Two pairs of guide RNAs (Supplementary Table 1) targeting the exon2 of human *CDKN2A* (NM_000077) were designed using ChopChop website. gRNA oligos were synthesized, annealed and subcloned into the pSpCas9(BB)-2A-GFP (PX458). G401 and A2780 cell lines were transfected with the resultant plasmids, cultured for 48 h, and then applied to the single cell sorting for selecting GFP-positive cell by flow cytometer. Single cell clones were first cultured each well of 96-well plate, and then gradually expanded in larger cultures for further experiments. We amplified the genomic DNA fragment through PCR using primers p16-cas9-primer-F and -R (Supplementary Table 1) for sequencing, and the positive clones were further validated using western blot of p16 in the presence of MAK683 treatment for 3–6 days.

### Western blotting

Cells were washed once with cold PBS and lysed by RIPA lysis buffer and incubated with 4 × SDS loading buffer at 95 °C for 10 min. Whole cell proteins were used for electrophoresis and then transferred to a nitrocellulose membrane. The membrane was blocked with Tris buffered saline containing 0.075% Tween-20 and 5% non-fat milk. Then the membranes were incubated with primary antibodies overnight at 4 °C. The next day, membranes were incubated with secondary antibodies for 1 h at room temperature.

Antibodies used in this study included H3 (Cell Signaling Technology, 9715; dilution 1:1000), H3K27me3 (Cell Signaling Technology, 9733; dilution 1:1000), H3K27me2 (Cell Signaling Technology, 9728; dilution 1:1000), H3K27me1 (Merck Millipore, 07-448; dilution 1:1000), H3K27ac (Cell Signaling Technology, 8173; dilution 1:1000), H3K4me3 (Merck Millipore, 04-745; dilution 1:1000), H3K36me3 (Cell Signaling Technology, 4909; dilution 1:1000), H3K9me3 (Active Motif, 39161; dilution 1:1000), H3K79me2 (Abcam, ab3594; dilution 1:1000), p16 (Santa Cruz, sc-377412; dilution 1:200), β-actin (Cell Signaling Technology, 3700, dilution 1:1000) (Supplementary Table 1).

### Chromatin immunoprecipitation (ChIP)

We used the ChIP kit from Millipore for ChIP-seq sample preparation (with spike-in step) and ChIP-qPCR analysis. Briefly, G401 cells (1 × 10^6^) were seeded in 10-cm dishes and treated with DMSO or 3 μM of MAK683 for 3 days. Cells were fixed with 0.7% formaldehyde in culture medium for 7 min at room temperature and the reaction was quenched with 0.125 M glycine sharply. Then remove media and wash the cell twice with cold PBS including protease inhibitor mix. Cells were scraped off the plate and the pellets were collected, lysed at the volume of 4 × 10^6^ per 300 μL using SDS lysis buffer for 10 min on ice, and then proceed to sonication. Each sample was sonicated for 14 min, 15 s on and 45 s off with 70% output (Qsonica 4905 Chiller). Sonicated samples were diluted with ChIP dilution buffer, additionally add 40 ng Drosophila chromatin for spike in normalization, and applied to 1 h pre-clear with 30 μL protein A+G agarose beads. After that, input samples were collected, and every 300 uL of cell lysate samples were applied into ChIP with 2 μg H2Av antibody plus 8 μg of H3K4me3 antibody, 8 μg of H3K27me3 antibody, or negative control lgG respectively. Antibody-chromatin complex were incubated overnight at 4 °C, then captured by 60 μL protein A+G agarose beads for 1 h at 4 °C. The beads were washed in low salt buffer once, high salt buffer once, LiCl buffer once, and TE buffer twice. DNA-protein complex was eluted twice with elution buffer (1% SDS, 0.1 M NaHCO_3_), eluted chromatin was de-crosslink at 65 °C for 4 h with 5 M NaCl and Proteinase K. Reserved chromatin DNA were purified with Phenol-chloroform method.

### RNA-seq data analysis

Total pair-end sequences obtained on Illumina PE150 (150-bp reads) instruments were aligned to the human genome (hg19). We used Trim-galore (version 0.4.4_dev) (Krueger F. Trim galore. 2012. Available from: http://www.bioinformatics.babraham.ac.uk/projects/trim_galore/) to remove low quality sequences as well as sequencing connectors. mRNA levels of genes in triplicate samples were calculated as FPKM using RSEM (version v1.3.0) [69] and featureCounts (version v1.5.3) [70]. We determined differential gene expression using R package DESeq2 (version v1.20.0) [71] with an FDR threshold of 0.05 and an Log2 Fold Change threshold of ±1.

### ChIP-seq and ATAC-seq data analysis

Total pair-end sequences obtained on Illumina PE150 (150-bp reads) instruments were aligned to the human genome (hg19). We used Trim-galore (version 0.4.4_dev) to remove low quality sequences as well as sequencing connectors. Sequences were aligned with Bowtie2 (version 7.3.0) [72]. ChIP-seq sample depleted repeat sequences by Picard (version 2.23.6) (“Picard Toolkit” 2019. Broad Institute, GitHub Repository. http://broadinstitute.github.io/picard/; Broad Institute) and normalized by drosophila histone modification (Spike-in). These reads were used to generate binding site with MACS2 (version 2.2.7.1) [73] and featureCounts (version v2.0.0).

#### Spike-in normalization

All samples were sequenced with corresponding Drosophila histone modification sequences and aligned to the Drosophila genome (dm6). Firstly, counted the number of read segments specifically matched to the Drosophila genome after remove duplication. Secondly, calculated standardization factor (*SF*) with the minimum number of reads matched to the Drosophila genome divided by the number of reads matched to the Drosophila genome of the corresponding samples.

$$SF_i = \frac{{Min\left( {UMD_1,UMD_2,UMD_3, \cdots ,UMD_i} \right)}}{{UMD_i}}$$

*UMD* represents the number of read segments specifically matched to Drosophila, *i* represents the *i*^*th*^ sample. Thirdly, multiplied the number of read segments matched to the human genome by the corresponding standardized factor.

$$NH_i = SF_i \ast OH_i$$

*NH* represents the number of read segments specifically matched to the human genome after standardization, *OH* represents the number of read segments specifically matched to the human genome before standardization, *i* represents the *i*^*th*^ sample.

### Bisulfite-seq data analysis

Total pair-end sequences obtained on Illumina NoveSeq 6000 PE150 (150-bp reads) instruments were aligned to the human genome (hg19). We used Trim-galore (version 0.4.4_dev) to remove low quality sequences as well as sequencing connectors. Sequences were aligned with Bismark (version v0.19.0) [74], which based on Bowtie2 (version 2.2.8) and can transformed reference genome by three-letter alignment.

### K-means cluster

#### Preprocessing

Due to different scales among different sequence features, z-score normalization was performed for all features.

#### Parameter selection

The optimal number of clusters was chosen using the elbow method. Through several iterations and calculating the contour coefficient of each cluster, the turning point of decreasing contour coefficient is determined as the number of clusters.

#### Implementation

All calculations are performed in Python (version 3.7.0) and its modules, mainly through the Cluster and Preprocessing methods of the Sklearn module (version 0.21.2) [75].

### Enrichment analysis

#### Fisher-exact test

Through Python (version 3.7.0) and its Scipy (version 1.2.1) module [76], the enrichment level of each gene set was calculated according to Fisher’s exact test method.

#### Gene set enrichment analysis (GSEA)

GSEA is based on GSEA Desktop Application (version 4.0.3, download from http://www.gsea-msigdb.org/gsea/downloads.jsp).

#### Database

All gene sets download from Molecular Signatures Database (MSigDB, http://www.gsea-msigdb.org/gsea/msigdb/index.jsp, version v7.1/v7.2), including C2.cp/C5.go gene sets.

### Visualization

Data visualization was performed using Python’s Matplotlib (version 3.1.0) [77] and Seaborn (version 0.9.0) [78] module, R’s Gviz (version 1.30.3) package [79] and DeepTools software (version 3.4.3) [80].

### Quantitative PCR

Total RNA samples were prepared using Trizol reagent (Sigma) following the protocol provided with the reagent. 2 μg of total RNA were reserve-transcribed to cDNA using M-MLV Reverse Transcriptase (Promega). Following cDNA synthesis, real-time quantitative PCR was performed using SYBR Green gene expression assay on CFX Real-time System (Bio-Rad). GAPDH was used as the normalization control, and the data were further calculated by the 2^−ΔΔCt^ method. All reactions were performed in triplicates. The sequence information for the primers are listed in Supplementary Table 2.

### Mouse xenograft studies

The animal study design and performance were evaluated and approved by Institutional Animal Care and Use Committee of ShanghaiTech University under the document number of 20201225001 and following the internationally recognized guidelines on animal welfare. To establish G401 xenograft models, WT G401 or p16 knock out cells were washed and resuspended in cold PBS and mixed with Matrigel at 1:1 (v/v) to reach the final ratio of 5 × 10^6^ cells per 200 μL. Then the tumor cell mix were injected subcutaneously into the right flank of female Balb/c nude mice (5–6 weeks of age).

When tumor volume reached around 60-350 mm^3^, the mice were numbered and randomized into vehicle or 100 mg/kg MAK683 treatment groups. The random numbers were generated using Excel (Software). The mice with tumor size out of the range were not included in the study. Empirically, at least 6 mice were included for each group. For compound dosing, MAK683 was resolved and suspended in water with 0.5% HPMC and 0.5% Tween at the concentration of 10 mg/mL. Dosing without blinding took place very day in the morning around 8 am. Mice weight and tumor volumes are measured and recorded every 3 days. Tumor volumes were calculated based on perpendicular length and width caliper measurements using the following formula: tumor volume (mm^3^) = 0.5 × (length × width^2^).

### Immunohistochemistry

Tumor tissues were fixed in freshly-prepared 4% PFA for 24 hours, dehydrated through 30%, 50%, 70%, 85%, and 90% ethanol five-minute each, and 100% ethanol triple, permeabilized through xylene five-minute twice and then embedded in paraffin, and finally cut into 5 μm-thick sections. The sections were de-paraffinized with xylene for 10 min triple, rehydrated with ethanol from high to low concentration five minutes each and dH_2_O twice. For antigen retrieval, sections were boiled in a microwave for 15 min in 10 mM citrate buffer (pH 6.0) for Ki-67 (Abcam, ab16667; dilution 1:100), H3K27me3 (Cell Signaling Technology, 9733; dilution 1:200), and F4/80 (Thermo Fisher, 14-4801-82; dilution 1:50); or in 10 mM Tris-EDTA buffer (pH 9.0) for ITGA2 (Abcam, ab181548; dilution 1:50) (Supplementary Table 1). After cool down to room temperature, endogenous peroxidase was inactivated with 3% H_2_O_2_ in methanol. Then the sections were washed with dH_2_O twice, blocked with 5% normal goat serum in PBS with 0.25% Triton X-100 for 1 hour at room temperature followed by primary antibodies incubation overnight at 4 °C. After HRP-conjugated secondary antibodies were incubated for 1 hours at room temperature, the sections were visualized with DAB. For quantification of Ki-67, H3K27me3 or ITGA2 positivity, the ratios of DAB positive cells to total cells in five representative fields were counted. For quantification of F4/80 positivity, the ratios of DAB positive area to total cells were counted. Five 40 × objective magnification fields per selection were counted and averaged.

### β-gal staining

Cell senescence study was used by Beyotime in Situ β-galactosidase Staining Kit. Adherent cells were fix by fix-buffer for 15 min at room temperature and wash with PBS twice, then stain by stain-buffer mixture at 37 °C overnight. Frozen tissue sections were counterstained with eosin.

### Statistical analysis

Statistical analysis for qPCR and tumor tissue section staining were carried out using GraphPad Prism 8 (Supplementary Table 1). Data are shown as mean ± s.d. The variance within each group is similar.

Statistical significance was determined using the two-tailed Student’s test (*t* test). *P* < 0.05 was considered significant.

## Supplementary information

Supplementary Figures

Supplementary table 1

Supplementary table 2

Reproducibility checklist

## Data Availability

The raw data files for the sequencing analysis that generated in this study are deposited in the NCBI Gene Expression Omnibus (GEO) under the accession number GSE183605. This accession number is the superseries including GSE183600 (RNA-seq), GSE183601 (ChIP-seq), GSE183602 (ATAC-seq).
