# Supplementary material for: Induction of senescence-associated secretory phenotype underlies the therapeutic efficacy of PRC2 inhibition in cancer
Source: Cell Death Dis. 2022 Feb 15;13(2):155. doi: 10.1038/s41419-022-04601-6 (PMC8847585; doi:10.1038/s41419-022-04601-6)
Supplement: Supplementary file 1 — Supplementary Figures [file 41419_2022_4601_MOESM1_ESM.docx]

**Induction of senescence-associated secretory phenotype underlies the therapeutic efficacy of PRC2 inhibition**

Liping Chu^1, 2, #^, Yuxiu Qu^1, #^, Yang An^1, #^, Linjun Hou^1^, Juewan Li^1^, Weijia Li^1^, Gaofeng Fan^1^, Bao-Liang Song^2^, En Li^3^, Liye Zhang^1^, Wei Qi^1, *^

^1^ School of Life Science and Technology, ShanghaiTech University, Shanghai, 201210, China

^2^ Hubei Key Laboratory of Cell Homeostasis, College of Life Sciences, Wuhan University, Wuhan, China

^3^ China Novartis Institutes for BioMedical Research, 4218 Jinke Road, Shanghai 201203, China

#: These authors contribute equally

*: Correspondence to: [qiwei@shanghaitech.edu.cn](mailto:qiwei@shanghaitech.edu.cn)

**Supplementary Methods**

**Patient data analysis**

*Preprocessing.* The data was downloaded from the GEO database (GSE62564). Convert the transcript name to the gene name, and combine the different transcripts of the same gene with the maximum value.

*Grouping.* The 25% samples with the lowest EZH2 expression level were divided into the low EZH2 group (126 samples), and the 25% samples with the highest EZH2 expression level were divided into the high EZH2 group (125 samples).

*Differential expression analysis*. For each gene, the expression fold change between the low EZH2 expression group and the high EZH2 expression group was calculated. Kruskal nonparametric test was used to calculate the p values and Bonferroni method was used to correct the p values.

*Pathway enrichment analysis.* The c5-bp gene set was used for pathway enrichment analysis of the Up-DEG genes. The analysis method was described in the Enrichment Analysis section.

**Supplementary figures**


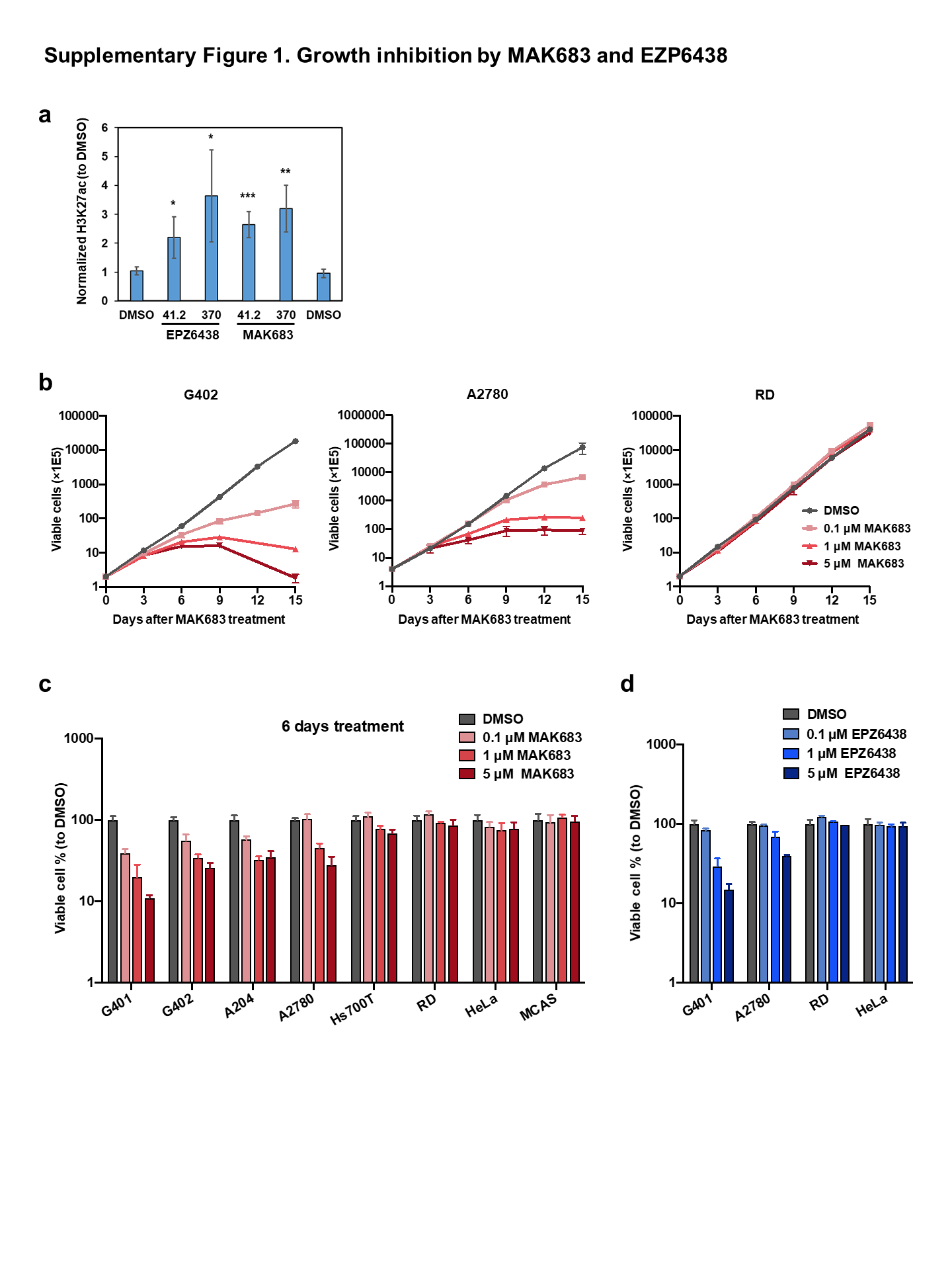


**Supplementary Fig. 1. Cancer cell growth inhibition by PRC2 inhibitors MAK683 and EPZ6438.**

1. Bar graph of the quantification of the western blot signal of H3K27ac upon treatment of EPZ6438 and MAK683 at the indicated concentrations (as samples in Fig. 1d, data are plotted as mean ± s.d. from five independent experiments).
2. Growth curve of rhabdoid cancer cells G402, A2780 and RD with DMSO or MAK683 at the indicated concentrations for the period of 15 days (n=2 or 3; mean ± s.d.).
3. Inhibition of the proliferation of multiple cells including G401, G402, A204, A2780, Hs700T, RD, HeLa and MCAS after 6 days of MAK683 treatment. Viable cells were counted every 3 days in the presence of MAK683 at the indicated concentrations, and results were normalized to the control samples (DMSO as 100%) of respective cells and then plotted on a logarithmic scale (n=2 or 3; mean ± s.d.)..
4. Inhibition of the proliferation of G401, A2780, RD and HeLa after 6 days of EPZ6438 treatment. Viable cells were counted every 3 days in the presence of EZP6438 at the indicated concentrations, and results were expressed similarly as in panel **b**. (n=2 or 3; mean ± s.d.).

**
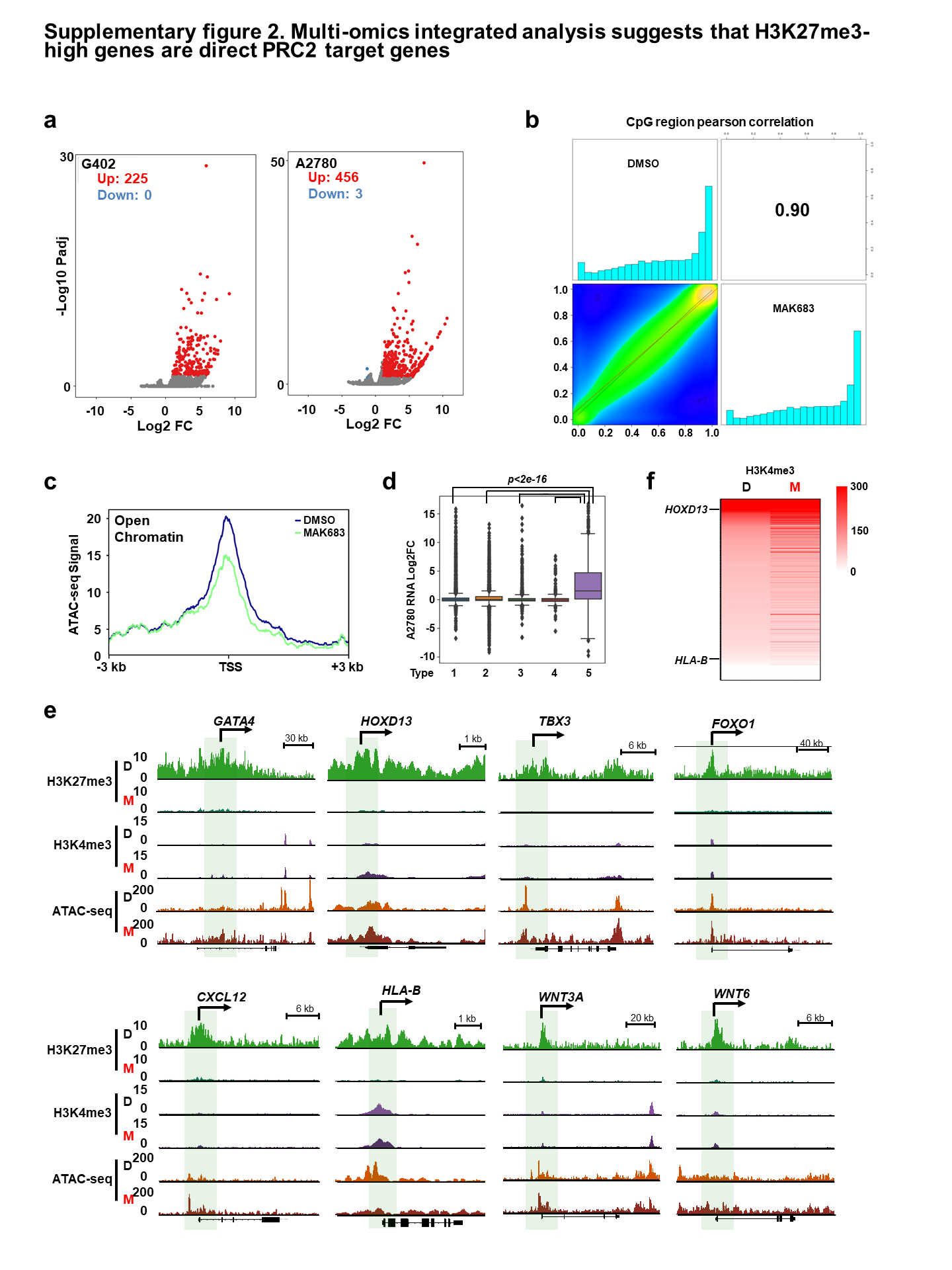
**

**Supplementary Fig. 2. Multi-omics integrated analysis suggests that H3K27me3-high genes are direct PRC2 target genes de-repressed by PRC2 inhibition**.

1. Volcano plot showing the differential expression genes (DEGs) of G402 and A2780 cells after DMSO, MAK683 treated for 3 days. Red shows up-regulated after MAK683 treatment (log2 fold change ≥ 1 and p adjust ≤ 0.05) and blue indicates down-regulated after MAK683 treatment (log2 fold change ≤ -1 and p adjust ≤ 0.05) unless otherwise noted.
2. Comparison of the DNA methylation profile of G401 cells with DMSO or MAK683 treatment at 3 μM for 6 days. The overall similarity between DMSO and MAK683 treated samples is 0.90.
3. Composite open chromatin profile around transcription starting sites (TSSs) extracted from ATAC-seq data in G401 cells with DMSO or MAK683 treatment.
4. Box plots of log2 mRNA fold change levels between different clustering types in A2780 cell in response to DMSO or MAK683 treatment. P values were determined by Kruskal nonparametric test.
5. H3K27me3 and H3K4me3 ChIP-seq tracks and ATAC-seq tracks at multiple gene loci in G401 cells treated with DMSO or MAK683. Green highlights indicated genomic regions around TSS.
6. Heat map of the H3K4me3 ChIP-seq signal of the 624 genes from panel Fig. 2g. *HOXD13* and *Hla-b* are shown as the benchmark bivalent genes with positive H3K4me3 signal.


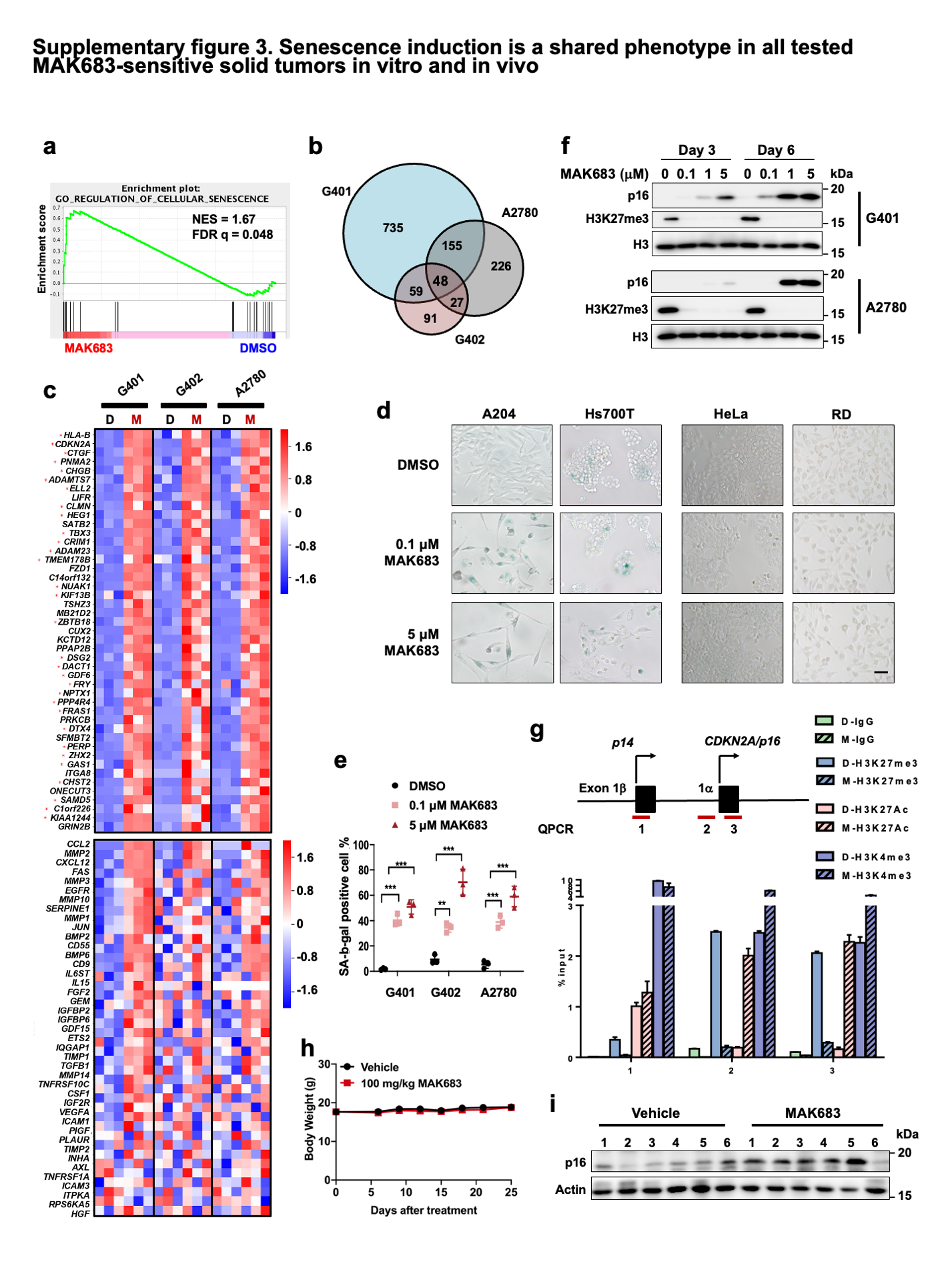


**Supplementary Fig. 3. Senescence induction is a shared phenotype in in multiple MAK683-responsive solid tumor cells with *SMARCB1* or *ARID1A* deficiency**.

1. GSEA of RNA-seq data from G401 cell with DMSO or MAK683 treatment as shown in Figure 2A revealing the enrichment of cellular senescence-related pathway.
2. Venn diagram showing the overlap of upregulated DEGs from RNA-seq analysis of the G401, G402 and A2780 cells after MAK683 treatment. The criteria for DEGs are log2 fold change ≥ 1 and p adjust ≤ 0.05.
3. Heat map of the 48 genes from panel **b** showing mRNA fold change levels from the RNA-seq data of G401, G402 and A2780 cells with DMSO or MAK683 treatment. The top panel shows the overlapped upregulated DEGs from the three MAK683-responsive cell lines without the three upregulated genes from RD. Red asterisks indicated genes showing upregulation in senescence induction conditions in SeneQuest database (<http://senequest.net/about>). The bottom panel shows the genes from the SASP list of Figure 3A. The color was coded according to Log2 (Fold Change + 0.1).
4. Representative senescence-associated β-galactosidase staining (SA-β-gal, blue) of A204, Hs700T, RD and HeLa cells treated with DMSO or MAK683 at the indicated concentrations for 9 days. RD and HeLa are not responsive to MAK683 and does not show positive staining. Scale bars represent 50 μm. Representative images of more than two independent experiments in the indicated cells.
5. Dot plots showing the quantification of the SA-β-gal positive cell percentage. Mean ± s.d. of positively stained cell percentage are shown. P values were determined by Multiple t test (**, p<0.01; ***, p<0.001).
6. Western blotting analysis of the G401 and A2780 cells treated with DMSO, 0.1 μM, 1 μM or 5 μM of MAK683 for 3 days or 6 days, showing the inhibition on H3K27me3 and upregulation of p16 protein levels in MAK683 treated cells.
7. ChIP-qPCR using anti-H3K27me3, H3K4me3 and H3K27Ac antibodies at the indicated regions on *CDKN2A/p16/* promoter before and after 72 hr of MAK683 treatment. Rabbit IgG is used as the control. Relative enrichment is calculated as percentage of input.
8. Effect of MAK683 treatment on the body weight of tumor-bearing animal as in study in Fig. 3f (n=6; mean ± s.e.m.).
9. Western blotting analysis of the tumor xenograft collected from the study in **a**, showing the upregulation of p16 protein levels in MAK683 treated tumors (n=6 for vehicle and MAK683 groups).


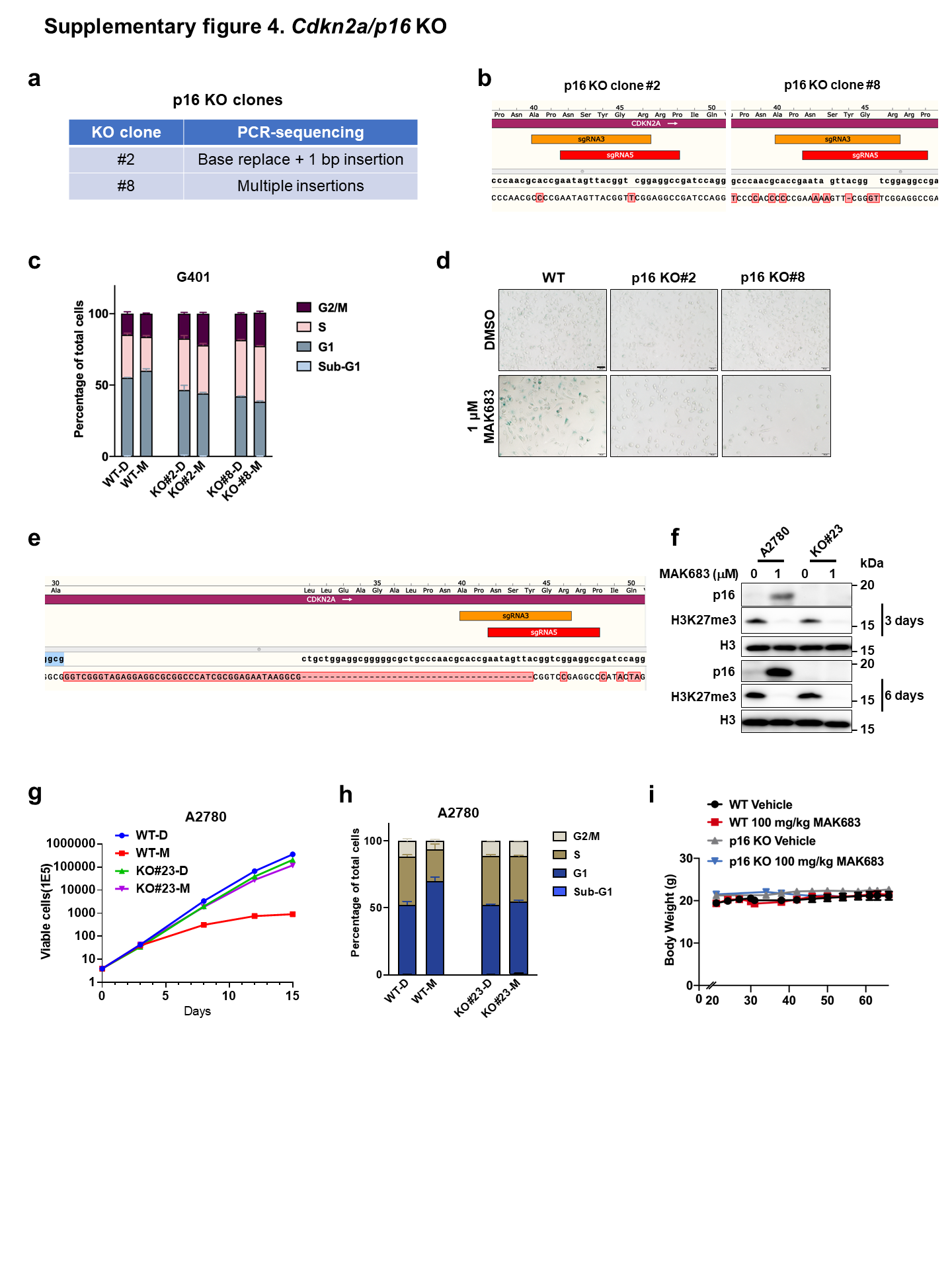


**Supplementary Fig. 4. Senescence induction is a shared phenotype in all tested MAK683-sensitive solid tumor cells**.

1. Table showing the genetic sequencing information of the two p16 KO G401 clones.
2. Sequence alignment showing the genetic changes of the *CDKN2A/p16* gene in the two p16 KO G401 clones.
3. Bar graph presenting the cell cycle changes of G401 WT and the two p16 KO clones analyzed by FACS after treatment with DMSO or 3 μM of MAK683 for 3 days (n=3, mean ± s.d.).
4. Representative SA-β-gal staining (blue) of G401 or p16 KO cells treated with DMSO or MAK683 at 1 μM for 9 days. Scale bars represent 50 μm. Representative images of more than two independent experiments in the indicated cells.
5. Sequence alignment showing the genetic changes of the *Cdkn2a/p16* gene in the p16 KO#23 A2780 clone.
6. Western blotting analysis showing the successful knockout of p16 in KO#23 A2780 cell clone, and the upregulation of p16 protein levels in MAK683 treated A2780 cell is shown as positive control. Inhibition on H3K27me3 by MAK683 in both WT A2780 and p16 KO#23 A2780 cells is similar.
7. Proliferation of A2780 and p16 KO#23 cells. Viable cells were counted every 3 days in the presence of MAK683 at 1 μM, and results were plotted on a logarithmic scale.
8. Bar graph presenting the cell cycle changes of A2780 and p16 KO#23 cells analyzed by FACS after treatment with DMSO or 3 μM of MAK683 for 3 days.
9. Effect of MAK683 treatment on the body weight of tumor-bearing animal as in study in Figure 4e (n=6; mean ± s.e.m.).

**
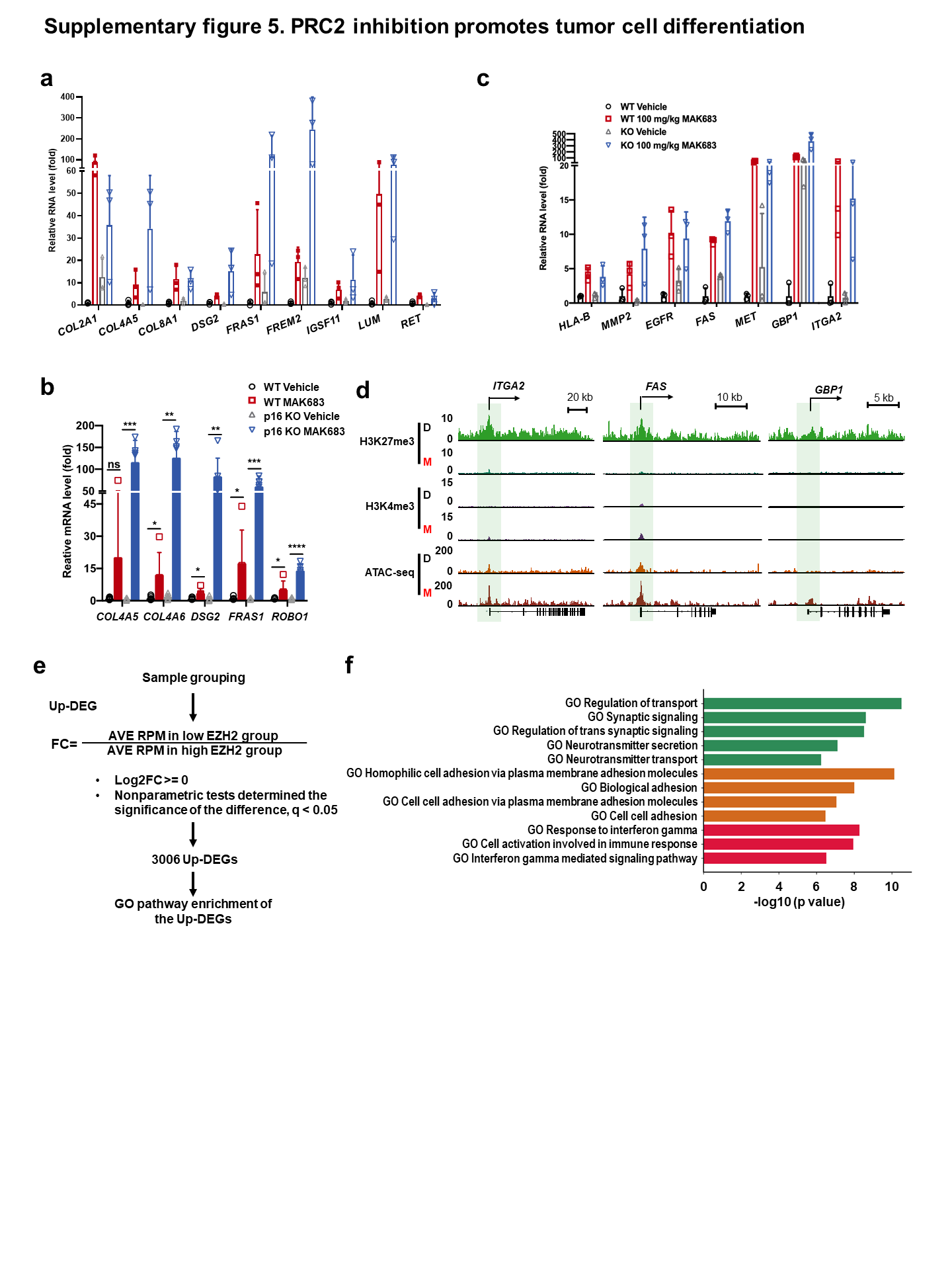
**

**Supplementary Fig. 5. PRC2 inhibition promotes tumor cell differentiation, senescence-associated inflammation and immune infiltration *in vivo.***

1. Bar graph showing the fold change of RNA-seq data for related genes in ECM pathway.
2. Quantitative PCR analysis of the expression changes of IFN-I response genes in G401 or p16 KO tumor samples after vehicle or MAK683 dosing (n=6; mean ± s.d.).
3. Bar graph showing the fold change of RNA-seq data for related genes in type I IFN and antigen processing and presentation (APP) pathways.
4. H3K27me3 and H3K4me3 ChIP-seq tracks and ATAC-seq tracks at multiple gene loci in G401 cells treated with DMSO or MAK683. Green highlights indicated genomic regions around TSS.
5. Outline of the procedures for the analysis of the neuroblastoma data to generate Up-DEGs in Figure 5H and GO pathway enrichment in panel F.
6. Pathway enrichment analysis of the Up-DEGs from the panel e. The green colored bar indicated the representative pathways related to synaptic signaling, the brown colored bar indicated the representative pathways related to matrix and cell adhesion, and the red colored bar indicated the representative pathways related to interferon pathways.
