# Supplementary material for: Induction of senescence-associated secretory phenotype underlies the therapeutic efficacy of PRC2 inhibition in cancer
Source: Cell Death Dis. 2022 Feb 15;13(2):155. doi: 10.1038/s41419-022-04601-6 (PMC8847585; doi:10.1038/s41419-022-04601-6)
Supplement: Supplementary file 2 — Supplementary table 1 [file 41419_2022_4601_MOESM2_ESM.docx]

**Supplementary table 1. The detailed information for reagents and resources**

| **REAGENT and RESOURCE** | **SOURCE** | **IDENTIFIER** |
| --- | --- | --- |
| **Antibodies** | | |
| Rabbit monoclonal Anti-H3K27me3 | Cell Signaling Technologies | cat#9733; RRID: AB_2616029 |
| Rabbit monoclonal Anti-H3K27me2 | Cell Signaling Technologies | cat#9728; RRID: AB_1281338 |
| Rabbit monoclonal Anti-H3K27me1 | Merck Millipore | cat#07-448; RRID: AB_310623 |
| Rabbit monoclonal Anti-H3K27ac | Cell Signaling Technologies | cat#8173; RRID: AB_10949503 |
| Rabbit monoclonal Anti-H3K4me3 | Merck Millipore | cat#04-745; RRID: AB_1163444 |
| Rabbit polyclonal Anti-H3K9me3 | Active Motif | cat#39161; RRID: AB_2532132 |
| Rabbit monoclonal Anti-H3K36me3 | Cell Signaling Technologies | cat#4909; RRID: AB_1950412 |
| Rabbit monoclonal Anti-H3K79me2 | Abcam | cat#ab3594; RRID: AB_303937 |
| Rabbit monoclonal Anti-H3 | Cell Signaling Technologies | cat#9715; RRID: AB_331563 |
| Rabbit monoclonal Anti-H3 (ChIP Formulated) | Cell Signaling Technologies | cat#4620; RRID: AB_1904005 |
| Mouse monoclonal Anti-p16 | Santa Cruz | cat#sc-377412 |
| Mouse monoclonal Anti-β-actin | Cell Signaling Technologies | cat#3700; RRID: AB_2242334 |
| Rabbit monoclonal Anti-ITGA2 | Abcam | cat#ab181548; RRID: AB_2847852 |
| Rabbit polyclonal Anti-GBP1 | Proteintech | 15303-1-AP |
| Rabbit monoclonal Anti-Ki-67 | Abcam | cat#ab16667; RRID: AB_302459 |
| Rat monoclonal Anti-F4/80 | Thermo Fisher | cat#14-4801-82; RRID: AB_467558 |
| **Critical Commercial Assays** | | |
| EpiTect Bisulfite Kit | Qiagen | cat#59104 |
| In Situ β-galactosidase Staining Kit | Beyotime | cat#C0602 |
| Propidium Iodide Solution | Sigma | cat#P4864 |
| Chromatin Immunoprecipitation (ChIP) Assay Kit | Merck/Millipore | cat#17-295 |
| **Oligonucleotides:** | | |
| shRNA targeting sequence: p16 #1  GGAGCAGCATGGAGCCTTCGG | N/A | N/A |
| shRNA targeting sequence: p16 #2  TGCCCAACGCACCGAATAGTTACGGTC | N/A | N/A |
| sgRNA targeting sequence: p16#1  CACCGAATAGTTACGGTCGG | N/A | N/A |
| sgRNA targeting sequence: p16#2  GGCCTCCGACCGTAACTATT | N/A | N/A |
| p16-cas9-primer-F: GGGTCGGGTAGAGGAGGTG | N/A | N/A |
| p16-cas9-primer-R: TGTGATTACAAACCCCTTCTGA | N/A | N/A |
| p16 ChIP-qPCR Primer1 left: GTGGGTCCCAGTCTGCAGTTA | Ito et al., 2018 | N/A |
| p16 ChIP-qPCR Primer1 right: CCTTTGGCACCAGAGGTGA | Ito et al., 2018 | N/A |
| p16 ChIP-qPCR Primer2 left: ACCCCGATTCAATTTGGCAG | Ito et al., 2018 | N/A |
| p16 ChIP-qPCR Primer2 right:  AAAAAGAAATCCGCCCCCG | Ito et al., 2018 | N/A |
| p16 ChIP-qPCR Primer3 left: AGAGGGTCTGCAGCGG | Ito et al., 2018 | N/A |
| p16 ChIP-qPCR Primer3 right: TCGAAGCGCTACCTGATTCC | Ito et al., 2018 | N/A |
| **Software and Algorithms** | | |
| Image J | N/A | https://imagej.nih.gov/ij/  RRID: SCR_003070 |
| GraphPad Prism | GraphPad software (V 8.4.2 (679)) | https://www.graphpad.com/scientificsoftware/  prism/RRID: SCR_002798 |
